# The third coronavirus epidemic in the third millennium: what’s next?

**DOI:** 10.3325/cmj.2020.61.1

**Published:** 2020-02

**Authors:** Rok Čivljak, Alemka Markotić, Ilija Kuzman

**Affiliations:** 1 Dr Fran Mihaljević University Hospital for Infectious Diseases, Zagreb, Croatia; 2 University of Zagreb School of Medicine, Zagreb, Croatia *rok.civljak@bfm.hr*; 3 Catholic University of Croatia, Zagreb, Croatia; 4 University of Rijeka School of Medicine, Rijeka, Croatia

The current epidemic of a new coronavirus disease (COVID-19), caused by a novel coronavirus (2019-nCoV), recently officially named severe acute respiratory syndrome coronavirus 2 (SARS-CoV-2), has reopened the issue of the role and importance of coronaviruses in human pathology (1-5). This epidemic definitively confirms that this heretofore relatively harmless family of viruses, *Coronaviridae,* includes major pathogens of epidemic potential. The COVID-19 epidemic has clearly demonstrated the power of infectious diseases, which have been responsible for many devastating epidemics throughout history. The epidemiological potential of emerging infectious diseases, especially zoonoses, is affected by numerous environmental, epidemiological, social, and economic factors (6,7). Emerging zoonoses pose both epidemiological and clinical challenges to health care professionals.

Since the 1960s, coronaviruses have caused a wide variety of human and animal diseases. In humans, they cause up to a third of all community-acquired upper respiratory tract infections, such as the common cold, pharyngitis, and otitis media. However, more severe forms of bronchiolitis, exacerbations of asthma, and pneumonia in children and adults have also been described, sometimes with fatal outcomes in infants, the elderly, and the immunocompromised. Some coronaviruses are associated with gastrointestinal disease in children. Sporadic infections of the central nervous system have also been reported, although the role of coronaviruses in infections outside the respiratory tract has not been completely clarified (8).

## Previous epidemics caused by coronaviruses

Most coronaviruses are adapted to their hosts, whether animal or human, although cases of possible animal-to-human transmission and adaptation have been described in the past two decades, causing two epidemics.

The first such outbreak originated in Guangdong, a southern province of the People’s Republic of China, in mid-November of 2002. The disease was named severe acute respiratory syndrome (SARS). The cause was shown to be a novel coronavirus (SARS-CoV), an animal virus that had crossed the species barrier and infected humans. The most likely reservoir were bats, with evidence that the virus was transmitted to a human through an intermediate host, probably a palm civet or raccoon dog (8,9).

In less than a year, SARS-CoV infected 8098 people in 26 countries, of whom 774 died (10,11). Approximately 25% of the patients developed organ failure, most often acute respiratory distress syndrome (ARDS), requiring admission to an intensive care unit (ICU), while the case fatality rate (CFR) was 9.6%. However, in elderly patients (>60 years), the CFR was over 40%. Poor outcomes were seen in patients with certain comorbidities (diabetes mellitus and hepatitis B virus infection), patients with atypical symptoms, and those with elevated lactic acid dehydrogenase (LDH) values on admission. Interestingly, the course of the disease was biphasic in 80% of the cases, especially those with severe clinical profiles, suggesting that immunological mechanisms, rather than only the direct action of SARS-CoV, are responsible for some of the complications and fatal outcomes (8,9).

Approximately 20% of the reported cases during this epidemic were health care workers. Therefore, in addition to persons exposed to animal sources and infected family members, health care workers were among the most heavily exposed and vulnerable individuals (9,10). During 2004, three minor outbreaks were described among laboratory personnel engaged in coronavirus research. Although several secondary cases, owing to close personal contact with infected patients, were described, there was no further spread of the epidemic. It is not clear how the SARS-CoV eventually disappeared and if it still circulates in nature among animal reservoirs. Despite ongoing surveillance, there have been no reports of SARS in humans worldwide since mid-2004 (11).

In the summer of 2012, another epidemic caused by a novel coronavirus broke out in the Middle East. The disease, often complicated with respiratory and renal failure, was called Middle East respiratory syndrome (MERS), while the novel coronavirus causing it was called Middle East respiratory syndrome coronavirus (MERS-CoV). Although a coronavirus, it is not related to the coronaviruses previously described as human pathogens. However, it is closely related to a coronavirus isolated from dromedary camels and bats, which are considered the primary reservoirs, albeit not the only ones (8,12).

From 2012 to the end of January 2020, over 2500 laboratory-confirmed MERS cases, including 866 associated deaths, were reported worldwide in 27 countries (13). The largest number of such cases has been reported among the elderly, diabetics, and patients with chronic diseases of the heart, lungs, and kidneys. Over 80% of the patients required admission to the ICU, most often due to the development of ARDS, respiratory insufficiency requiring mechanical ventilation, acute kidney injury, or shock. The CFR is around 35%, and even 75% in patients >60 years of age. However, MERS-CoV, unlike its predecessor SARS-CoV, did not disappear, but still circulates among animal and human populations, occasionally causing outbreaks, either in connection with exposure to camels or infected persons (12).

Overall, 19.1% of all MERS cases have been among health care workers, and more than half of all laboratory-confirmed secondary cases were transmitted from human to human in health care settings, at least in part due to shortcomings in infection prevention and control (12,13). Post-exposure prophylaxis with ribavirin and lopinavir/ritonavir decreased the MERS-CoV risk in health care workers by 40% (14).

## The emergence of COVID-19 caused by SARS-CoV-2

In mid-December of 2019, a pneumonia outbreak erupted once again in China, in the city of Wuhan, the province of Hubei (1). The outbreak spread during the next two months throughout the country, with currently over 80 000 cases and more than 2400 fatal outcomes (CFR 2.5%), according to official reports. Exported cases have been reported in 30 countries throughout the world, with over 2400 registered cases, of which 276 are in Europe. On February 25, the first case of COVID-19 was confirmed in Zagreb, Croatia, and was linked to the current outbreak in the Lombardy and Veneto regions of northern Italy (15).

The case definition was first established on January 10 and modified over time, taking into account both the virus epidemiology and clinical presentation. The clinical criteria were expanded on February 4 to include any lower acute respiratory diseases, and the epidemiological criterion was extended to the whole of China, with the possibility of expansion to some surrounding countries (16,17).

At the early stage of the outbreak, patients’ full-length genome sequences were identified, showing that the virus shares 79.5% sequence identity with SARS-CoV. Furthermore, 96% of its whole genome is identical to bat coronavirus. It was also shown that this virus uses the same cell entry receptor, ACE2, as SARS-CoV (18).

The full clinical spectrum of COVID-19 ranges from asymptomatic cases, mild cases that do not require hospitalization, to severe cases that require hospitalization and ICU treatment, and those with fatal outcomes. Most cases were classified as mild (81%), 14% as severe, and 5% as critical (ie, respiratory failure, septic shock, and/or multiple organ dysfunction or failure). The overall CFR was 2.3%, while the rate in patients with comorbidities was considerably higher – 10.5% for cardiovascular disease, 7.3% for diabetes, 6.3% for chronic respiratory diseases, 6.0% for hypertension, and 5.6% for cancer. The CFR in critical patients was as high as 49.0% (4).

It is still not clear which factors contribute to the risk of transmitting the infection, especially by persons who are in the incubation stage or asymptomatic, as well as which factors contribute to the severity of the disease and fatal outcome. Evidence from various types of additional studies is needed to control the epidemic (19).

However, it is certain that the binding of the virus to the ACE 2 receptor can induce certain immunoreactions, and the receptor diversity between humans and animal species designated as SARS-CoV-2 reservoirs further increases the complexity of COVID-19 immunopathogenicity (20). Recently, a diagnostic RT-PCR assay for the detection of SARS-CoV-19 has been developed using synthetic nucleic acid technology, despite the lack of virus isolates and clinical samples, owing to its close relation to SARS. Additional diagnostic tests are in the pipeline, some of which are likely to become commercially available soon (21).

Currently, randomized controlled trials have not shown any specific antiviral treatment to be effective for COVID-19. Therefore, treatment is based on symptomatic and supportive care, with intensive care measures for the most severe cases (22). However, many forms of specific treatment are being tried, with various results, such as with remdesivir, lopinavir/ritonavir, chloroquine phosphate, convalescent plasma from patients who have recovered from COVID-19, and others (23-26).

No vaccine is currently available, but researchers and vaccine manufacturers have been attempting to develop the best option for COVID-19 prevention. So far, the basic target molecule for the production of a vaccine, as well as therapeutic antibodies, is the CoV spike (S) glycoprotein (27,28).

The spread of the epidemic can be only contained, and SARS-CoV-2 transmission in hospitals reduced, by strict compliance with infection prevention and control measures (contact, droplet, and airborne precautions) (22,29). During the current epidemic, health care workers have been at an increased risk of contracting the disease and consequent fatal outcome owing to direct exposure to patients. Early reports from the beginning of the epidemic indicated that a large proportion of the patients had contracted the infection in a health care facility (as high as 41%), and that health care workers constituted a large proportion of these cases (as high as 29%). However, the largest study to date on more than 72 000 patients from China has shown that health care workers make up 3.8% of the patients. In this study, although the overall CFR was 2.3%, among health care workers it was only 0.3%. In China, the number of severe or critical cases among health care workers has declined overall, from 45.0% in early January to 8.7% in early February (4). This poses numerous psychological and ethical questions about health care workers’ role in the spread, eventual arrest, and possible consequences of epidemics. For example, during the 2014-2016 Ebola virus disease epidemic in Africa, health care workers risked their lives in order to perform life-saving invasive procedures (intravenous indwelling, hemodialysis, reanimation, mechanical ventilation), and suffered high stress and fatigue levels, which may have prevented them from practicing optimal safety measures, sometimes with dire consequences (30).

## A lesson for the future

This third coronavirus epidemic, caused by the highly pathogenic SARS-CoV-2, underscores the need for the ongoing surveillance of infectious disease trends throughout the world. The examples of pandemic influenza, avian influenza, but also the three epidemics caused by the novel coronaviruses, indicate that respiratory infections are a major threat to humanity. Although Ebola virus disease and avian influenza are far more contagious and influenza currently has a greater epidemic potential, each of the three novel coronaviruses require urgent epidemiologic surveillance. Many infectious diseases, such as diphtheria, measles, and whooping cough, have been largely or completely eradicated or controlled through the use of vaccines. It is hoped that developments in vaccinology and antiviral treatment, as well as new preventive measures, will ultimately vanquish this and other potential threats from infectious diseases in the future.
